# Pott-Puffy-Tumor: die Notwendigkeit einer interdisziplinären Diagnostik und Behandlung

**DOI:** 10.1007/s00106-021-01133-x

**Published:** 2022-03-08

**Authors:** Jan Philipp Kühn, Stefan Linsler, Nasenien Nourkami-Tutdibi, Sascha Meyer, Sören L. Becker, Umut Yilmaz, Bernhard Schick, Alessandro Bozzato, Philipp Kulas

**Affiliations:** 1 Klinik für Hals‑, Nasen- und Ohrenheilkunde, Universitätsklinikum des Saarlandes, Kirrberger Straße, 66421 Homburg, Deutschland; 2 Klinik für Neurochirurgie, Universitätsklinikum des Saarlandes, Homburg, Deutschland; 3 Klinik für Allgemeine Pädiatrie und Neonatologie, Universitätsklinikum des Saarlandes, Homburg, Deutschland; 4 Institut für Medizinische Mikrobiologie und Hygiene, Institute für Infektionsmedizin, Universitätsklinikum des Saarlandes, Homburg, Deutschland; 5 Klinik für diagnostische und interventionelle Neuroradiologie, Universitätsklinikum des Saarlandes, Homburg, Deutschland

**Keywords:** Osteomyelitis, Stirnbein, Frontaler subperiostaler Abszess, Kinder, Jugendliche, Osteomyelitis, Frontal bone, Frontal subperiosteal abscess, Children, Adolescents

## Abstract

Der Pott-Puffy-Tumor (PPT) stellt als bakterielle Infektion des Sinus frontalis mit subperiostaler und intrakranieller Abszessbildung eine seltene Erkrankung in der Pädiatrie dar. Nachfolgend präsentieren wir vier Fälle eines PPT, die bei zwei Kindern (6 und 9 Jahre) und bei zwei jungen Erwachsenen (17 und 19 Jahre) auftraten. Alle Patienten wurden interdisziplinär von einem Team aus Neurochirurgie, Pädiatrie, HNO-Heilkunde, Neuroradiologie und Mikrobiologie betreut. Die Antibiotikabehandlung wurde in einem Fall mit einer endoskopischen Nasennebenhöhlenoperation (FESS) und in den anderen drei Fällen zusätzlich mit einem offenen transkraniellen Zugang zur Drainage der intrakraniellen Abszessbildung kombiniert. Da der PPT im Kindesalter mit dem Befund einer intrakraniellen Abszessbildung einhergehen kann, ist eine enge interdisziplinäre Zusammenarbeit für eine erfolgreiche Behandlung dieser seltenen Erkrankung erforderlich.

## Abstract (en)

Pott’s puffy tumor (PPT) is an infection of the frontal sinus with subperiosteal and intracranial abscess formation and one of the rare entities in pediatrics. We present a series of four cases of PPT that occurred in two children (6 and 9 years) and in two young adults (17 and 19 years). All patients were treated by an interdisciplinary team of pediatric, neurosurgical, ENT, radiological, and neuroradiological specialists. Antibiotic treatment was combined with single endoscopic surgery in one case and combined endoscopic sinus surgery with an open transcranial approach to drain intracranial abscess formation in three cases. It is important to be aware that PPT occurs in children with the finding of intracranial abscess formation. Therefore, a close interdisciplinary cooperation for successful treatment is needed in this rare disease.

## Hintergrund

Der Pott-Puffy-Tumor (PPT) stellt ein seltenes Krankheitsbild dar, welches durch eine lokalisierte Schwellung der Stirn gekennzeichnet ist und erstmals von Sir Percival Pott im 18. Jahrhundert als Abszessbildung mit einem extraduralen Empyem im Zusammenhang mit einem frontalen Kopftrauma beschrieben wurde [7]. Meist tritt ein PPT nach einer unbehandelten bzw. unzureichend behandelten Sinusitis auf [2, 10], die sich dann über das Os frontale ausbreitet und zu einer Osteomyelitis mit extra- und intrakraniellen Abszedierungen führt [4]. Das Erregerspektrum ist analog einer „community-acquired“ Sinusitis mit Erregern wie Streptococcus spp., Staphylococcus spp., Haemophilus influenzae, Klebsiella spp., Anaerobier und Enterokokken, wobei Staphylokokken den häufigsten Erreger darstellten [2, 3]. Da viele dieser Erreger zur physiologischen Hautflora gehören, ist oftmals die klinische Relevanz bei einem Nachweis durch Abstriche und Gewebsproben schwierig einzuschätzen. Auftreten kann ein PPT in jedem Alter, wobei Jugendliche mit bereits entwickelter Stirnhöhle überdurchschnittlich häufig betroffen sind, was durch eine höhere Rate an Infektion der oberen Atemwege in diesem Alter begründet ist [9]. Die meisten Fälle von PPT betreffen gesunde Patienten, jedoch ist auch ein Auftreten nach Kokainabusus, dentalen Infektionen oder als Spätfolge von neurochirurgischen Interventionen beschrieben [3]. Die bei der Geburt noch nicht entwickelten Stirnhöhlen beginnen im Alter von ca. zwei Jahren zu pneumatisieren und sind im Jugendalter fast vollständig entwickelt. Die venöse Drainage der Stirnhöhle erfolgt durch diploische Venen, die mit den duralen venösen Sinus kommunizieren und möglicherweise so zur Entstehung von septischen Embolien beitragen [9]. Intrakranielle Komplikationen wie Duralvenenthrombose und Thrombosen des Sinus cavernosus, Meningitis, epidurale, subdurale oder intraparenchymale Abszesse treten aufgrund venöser Drainage oder direkter Ausbreitung auf. Als häufigste Symptome sind eitrige Rhinorrhö, Kopfschmerzen und frontale und periorbitale Schwellung, Fieber, Erbrechen sowie andere Anzeichen von Meningitis oder Enzephalitis zu nennen [11, 17]. Zur Planung der weiteren Behandlung kommt die Computertomographie (CT) und zur Diagnostik von intrakraniellen Komplikationen die Magnetresonanztomographie (MRT) zum Einsatz. Obwohl PPT meist eine Indikation zur chirurgischen Notfalltherapie darstellt, kann in ausgewählten Fällen auch ein rein konservativer Therapieansatz in Betracht gezogen werden.

Wir präsentieren in dieser Arbeit mehrere Fälle eines PPT, welche an in einem Zentrum der Maximalversorgung behandelt wurden.

## Fallbeispiele

### Patient 1

**Figure 1:**
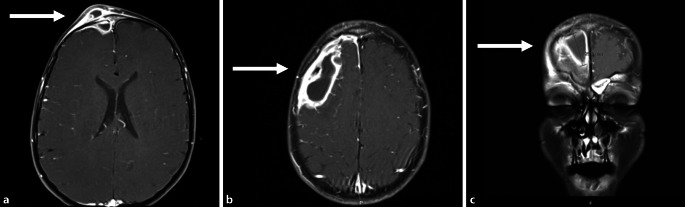

Ein 6‑jähriges Kind wurde uns aus der pädiatrischen Abteilung eines Krankenhauses aufgrund einer unklaren Schwellung der Stirn zugewiesen. Dort wurde das Kind bereits 6 Tage lang mit Ampicillin/Sulbactam bei der Verdachtsdiagnose einer Sinusitis frontalis behandelt. Nach einer kurzzeitigen Entlassung erfolgte die Wiedervorstellung aufgrund einer neuerlichen Schwellung über dem Sinus frontalis. Weitere Beschwerden oder Symptome lagen zum Zeitpunkt der Untersuchung nicht vor. Das Kind war fieberfrei, die Eltern berichteten jedoch über seit mehreren Wochen wiederkehrende Fieberepisoden sowie eine rezidivierende Sinusitis. Laborchemisch zeigte sich ein erhöhtes C‑reaktives Protein (CRP) von 61 mg/l (Normalbereich 0,0–5,0 mg/l) sowie eine Leukozytose von 20,6 × 10^9^/l (Normalbereich 4,8–12,0 × 10^9^/l). In der sonographischen Untersuchung der Stirn stellte sich eine inhomogene, echoarme Läsion mit einer Verdickung des darüber liegenden Gewebes zur Haut dar. Soweit sonographisch abbildbar, war das Os frontale intakt, ohne dass die Kortikalis unterbrochen war. Zur weiteren Diagnostik erfolgte im Anschluss eine MRT- sowie CT-Untersuchung. Hierbei zeigte sich jedoch eine Osteomyelitis des rechten Os frontale mit begleitenden subperiostalen sowie subduralen Abszessen und dem Verdacht auf eine beginnende Meningoenzephalitis. Darüber hinaus wurde eine Sinusitis frontalis beschrieben, wobei in der Zusammenfassung aller Befunde die Diagnose PPT gestellt wurde (Abb. 1). Zur Sanierung des Befundes erfolgte daraufhin eine frontolaterale Kraniotomie in Kombination mit einer funktionellen endonasalen Nasennebenhöhlenoperation (FESS). Hierbei wurde der affizierte Sinus frontalis eröffnet, wobei der vorbeschriebene intrakranielle, epidurale und subdurale Abszess drainiert werden konnte. Bei der endonasalen Inspektion zeigten sich keine weiteren Abszessformationen in den übrigen Nasennebenhöhlen. Nach erfolgreicher Drainage gestaltete sich der weitere Verlauf des Patienten regelrecht. In den mikrobiologischen Kulturen konnte ein Streptococcus intermedius nachgewiesen werden, und die bereits begonnene Antibiotikabehandlung wurde gemäß dem Resistogramm auf Cefotaxim und Clindamycin umgestellt und für insgesamt 14 Tage fortgeführt. Nach diesem Zeitraum konnte der Patient in gutem Allgemeinzustand ohne neurologische Defizite entlassen werden.

### Patient 2

**Figure 2:**
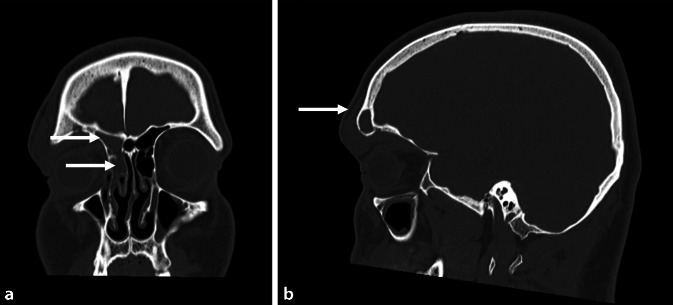

Ein 19-jähriger männlicher Patient stellte sich mit einer akuten Schwellung im Bereich des rechten Orbitarands sowie der Stirn vor. Extern war 20 Tage zurvor bereits eine FEES aufgrund einer rechtsseitigen Stirnhöhlenpyozele mit begleitender orbitaler Komplikation und einer analogen Symptomatik durchgeführt worden. Eine Visus- und Mobilitätseinschränkung des rechten Auges konnte ophthalmologisch ausgeschlossen werden. Symptome wie Fieber oder Schmerzen wurden vom Patienten verneint. Die laborchemische Untersuchung ergab ein CRP von 9,6 mg/l und Leukozyten von 9,9 × 10^9^/l. Die CT-Untersuchung zeigte eine Osteomyelitis mit einer geringgradigen frontalen Erosion der knöchernen Schädelbasis (Abb. 2). Eine konservative Behandlung wurde mit einer hochdosierten intravenösen Antibiotikatherapie mit Ceftriaxon 4g (1-0-0) eingeleitet. Unter dieser Therapie besserten sich die Symptome signifikant, und der Patient konnte nach vier Tagen ambulant weiter behandelt werden. Acht Tage später stellte sich der Patient erneut mit nun progredienten Symptomen und einer erneuten Schwellung im Bereich der rechten Orbita sowie Stirn vor. In der durchgeführten CT-Untersuchung zeigte sich ein periorbitaler Abszess auf Höhe der lateralen Augenbraue rechts mit einer Ausdehnung nach intraorbital sowie in den Sinus frontalis. Aus diesem Grund erfolgte ein Revisionseingriff im Sinne einer transnasalen Stirnhöhlenoperation Typ IIc (Typ-IIb-Drainage nach Draf in Verbindung mit einer Resektion des Septum interfrontale ohne Eröffnung des gegenseitigen Recessus frontalis) sowie eine transorbitale Abszessspaltung. In den mikrobiellen Kulturen wurde ein S. intermedius nachgewiesen und somit die Antibiotikatherapie nach Vorliegen des Resistogramms mit Ceftriaxon fortgesetzt. Nach 5 Tagen konnte der Patient in einem guten Allgemeinzustand sowie ohne Einschränkung des Visus und mit einer vollständigen Regredienz der frontalen Schwellung entlassen werden. Der weitere poststationäre Verlauf zeigte sich regelhaft.

### Patient 3

**Figure 3:**
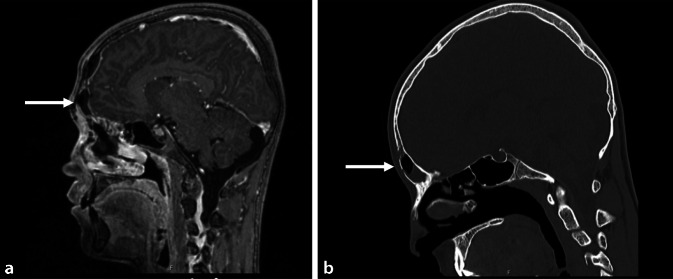

Ein 17-jähriger männlicher Patient stellte sich mit seit zwei Tagen bestehendem Fieber, Husten sowie Zephalgien vor. Initial war bereits eine ambulante symptomatische Behandlung im Sinne einer antipyretischen sowie analgetischen Therapie begonnen worden. Aufgrund einer jetzt akut aufgetretenen frontalen Schwellung erfolgte die Vorstellung in der Klinik für Hals‑, Nasen- und Ohrenheilkunde. Die Laborergebnisse zeigten ein stark erhöhtes CRP von 273,3 mg/l sowie eine Leukozytose von 15 × 10^9^/l. Bei der bereits klinischen Verdachtsdiagnose auf einen PPT erfolgten eine MRT- wie auch CT-Bildgebung, in welchen der klinische Verdacht bestätigt werden konnte. Zusätzlich zu den sinufrontalen Herden zeigten sich in der CCT zwei epidurale Abszesse im Bereich des Os frontale mit einer Verbindung zum linken Sinus frontalis und einer Ausdehnung von 8 × 9 mm sowie 14 × 9 mm (Abb. 3). Ein intrazerebraler Abszess konnte ausgeschlossen werden. Nach Einleitung einer Antibiotikatherapie mit Ceftriaxon wurde umgehend eine endonasale operative Stirnhöhlendrainage durchgeführt. Der subkutane präfrontale Abszess wurde über einen Schnitt im Bereich der Korrugatorfalte drainiert. Bei der MRT- und CT-Kontrolle am Folgetag waren die epiduralen Abszessformationen weiterhin bestehend, sodass von neurochirurgischer Seite eine transfrontale Entlastung der epiduralen Abszesse vorgenommen wurde. Der Patient erholte sich ohne neurologische Folgen und wurde nach einer abstrichgerechten zweiwöchigen Therapie mit Metronidazol und Ceftriaxon in gutem Allgemeinzustand entlassen.

### Patient 4

**Figure 4:**
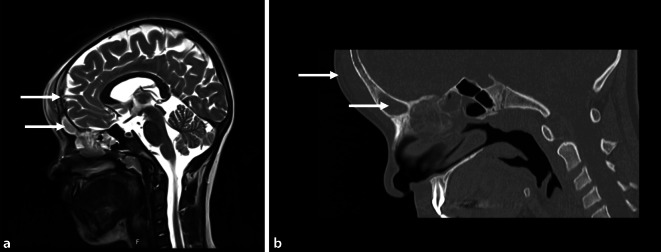

Ein 9‑jähriges Mädchen wurde etwa 6 Wochen lang mit anhaltenden und fortschreitenden Erkältungssymptomen im Krankenhaus behandelt. Eine Antibiotikabehandlung führte nicht zu einer relevanten Verbesserung der Symptomatik sowie des Allgemeinzustands des Kindes. Bei der ersten Vorstellung beschrieben die Eltern des Kindes rezidivierende Fieberepisoden bis zu 40 °C und eine neu entwickelte Schwellung der Stirn. Die Laborergebnisse zeigten ein leicht erhöhtes CRP von 30,3 mg/l und eine Leukozytose von 10,1 × 10^9^/l. Die daraufhin durchgeführten MRT- und CT-Untersuchungen zeigte eine Sinusitis mit einer frontalen Osteomyelitis, assoziiert mit einer subgalealen und epiduralen Abszessformation, jedoch ohne intrazerebrale Beteiligung (Abb. 4). Daraufhin erfolgte eine kombinierte HNO‑/neurochirurgische Intervention im Sinne einer endoskopischen Nasennebenhöhlenoperation in Verbindung mit einer osteoplastischen Kraniotomie und Drainage des epiduralen Abszesses sowie Resektion und Abfräsung des osteomyelitischen Knochens. Die zunächst empirisch indizierte Antibiotikatherapie mit Cefotaxim und Clindamycin wurde nach dem Nachweis von S. intermedius auf Ampicillin/Sulbactam umgestellt und für 14 Tage fortgeführt. Die epidurale Abszessbildung und die Schwellung des frontalen Weichgewebes waren im Rahmen der klinischen Kontrollen vollständig regredient, sodass das Kind ohne neurologische Folgen nach 14 Tagen in die Häuslichkeit entlassen werden konnte.

## Diskussion

### Inzidenz im Jugendalter

Wie zuvor beschrieben, treten die meisten Fälle von PPT im Jugendalter auf, was zum einen durch die sich in diesem Alter noch in Entwicklung befindlichen Stirnhöhlen und zum anderen durch die erhöhte Rate an Infekten der oberen Atemwege in diesem Alter begünstigt wird [9, 11]. Des Weiteren nimmt der Durchmesser und die Flussrate in den Diploe-Venen, die die Stirnhöhlen drainieren, im Jugendalter zu, was die hämatogene Ausbreitung von Infektionen in den Knochen und nach intrakraniell fördert [6, 21]. Darüber hinaus ist der Schädelknochen, der die Stirnhöhle begrenzt, bei Jugendlichen viel dünner als bei Erwachsenen, sodass zwischen der Infektion der Stirnhöhle und dem Knochen eine kürzere Distanz besteht, was die direkte sowie hämatogene Ausbreitung von Erregern begünstigt [21].

Die Pneumatisierung der Stirnhöhlen ist meist erst im Alter von 14 bis 15 Jahren abgeschlossen. Bei der Entwicklung des Nasennebenhöhlensystems wachsen die Stirnhöhlen hierbei kontinuierlich aus den lufthaltigen Siebbeinzellen in kranialer Richtung [12, 19]. Dies erklärt die Tatsache, dass jüngere Kinder aufgrund unvollständiger oder weniger pneumatisierter Stirnhöhlen weniger wahrscheinlich an einem PPT erkranken. Die vorgestellten Fälle zeigen jedoch, dass auch jüngere Kinder im Alter von 6 und 9 Jahren eine entsprechende Symptomatik entwickeln können (Tab. 1); obwohl die Nebenhöhlen noch nicht vollständig ausgebildet sind, können Infektionen des vorderen Siebbeins in diesen Fällen ihren Weg über das Siebbein in den Bereich der späteren Stirnhöhle finden.

**Table 1:** 

Patient	Bakterienspektrum	Antibiotische Therapie	Operative Behandlung
**Patient 1** **6 Jahre, ♂**	*Streptococcus intermedius*	Sultamicillin und Clindamycin	FESS in Kombination mit einer frontolateralen Kraniotomie
**Patient 2** **19 Jahre, ♂**	*Streptococcus intermedius*	Ceftriaxon	FESS in Kombination mit einem äußeren Zugang zur Orbita
**Patient 3** **17 Jahre, ♂**	*Eikenella corrodens*, *Staphylococcus capitis*	Metronidazol und Ceftriaxon	FESS in Kombination mit einer Entlastung über der Korrugatorfalte und transfrontaler Kraniotomie
**Patient 4** **9 Jahre, ♀**	*Streptococcus intermedius*, *Corynebacterium* spp.	Ampicillin und Sulbactam	FESS in Kombination mit einer frontolateralen Kraniotomie

*FESS* funktionelle endonasale Nasennebenhöhlenoperation

### Pathogenese

Auf dem Boden einer Entzündung der Cellulae ethmoidales oder des Sinus frontalis können sich Erreger über Diploe-Venen ausbreiten oder bei Affektion des Knochens direkt zur Demineralisierung mit Nekrose und so zur Entstehung einer Osteomyelitis führen [23]. Die Ausbreitung der Infektion wird durch das Fehlen von Venenklappen in den Diploe-Venen weiter gefördert. Des Weiteren kann sich ein Infekt über eine retrograde Thrombophlebitis ausbreiten, wobei bei Destruktion der Vorderwand der Stirnhöhle ein subperiostaler und bei Zerstörung der Hinterwand ein epiduraler Abszess entstehen kann. Ist die kaudale Wand des Sinus frontalis betroffen, kann sich die Infektion auf die Orbita ausbreiten und zu einer orbitalen Komplikation, beginnend mit einer präseptalen Zellulitis bis zu einem orbitalen Abszess und einer Phlegmone, führen.

### Diagnose

Die Diagnose des PPT wird zumeist bereits während der klinischen Untersuchung gestellt. In vielen Fällen zeigen die Patienten eine frontale Schwellung im Bereich des Sinus frontalis, welche teilweise die Orbita erreicht, oftmals assoziiert mit Zephalgien, klarer oder eitriger Rhinorrhö und/oder Fieber [2, 8]. Als Differenzialdiagnose einer solchen Stirnschwellung ist neben der akuten Sinusitis an ein infiziertes Atherom, ein Karbunkel oder ein Hämatom, z. B. bei Zustand nach einem Kopftrauma, zu denken [12, 20]. Das Blutbild ist in vielen Fällen von einer Leukozytose und erhöhten BSG- und CRP-Werten geprägt [1]. Die Anamnese eines Kopftraumas oder einer akuten sowie chronischen Sinusitis kann bei der Diagnosefindung hilfreich sein. Bei einer intrakraniellen Beteiligung können die Patienten auch Befunde eines intrakraniellen Druckanstiegs zeigen, der durch Übelkeit, fokale oder neurologische Defizite bis hin zu Bewusstlosigkeit gekennzeichnet ist [2, 15]. Eine Ultraschalluntersuchung kann in der Differenzierung der Stirnschwellung wichtige Hinweise geben. Hierbei kann zum einen zwischen einer soliden oder liquiden Schwellung unterschieden werden, und es können die Strukturen des Os frontale genauer beurteilt werden [16]. Als weniger invasive Methode sollte der Ultraschall besonders für Kinder vor der MRT- und/oder CT-Untersuchung eingesetzt werden [18]. Um jedoch in letzter Konsequenz die Destruktion des Os frontale und eine subperiostale Flüssigkeitssammlung zu diagnostizieren, wird eine kontrastmittelverstärkte CT-Untersuchung als Goldstandard angesehen [5].

Darüber hinaus ist die CT vor dem chirurgischen Eingriff von entscheidender Bedeutung, um differenzialdiagnostisch neben der Affektion der Stirnhöhlen eine Beteiligung weiterer Nasennebenhöhlen oder beispielsweise der Orbita zu diagnostizieren [11, 14]. Bei Verdacht auf eine intrakranielle und/oder intrazerebrale Beteiligung sollte des Weiteren eine MRT rechtzeitig durchgeführt werden, um die Notwendigkeit einer intrakraniellen Intervention frühzeitig planen zu können. Zusätzlich zu den Meningen können so auch die intraorbitalen Strukturen und eine Sinusvenenthrombose ausgeschlossen oder diagnostiziert werden [11]. Zur Planung eines operativen Eingriffs, insbesondere im Hinblick auf eine transnasale Drainage des Fokus im Sinne einer FESS, sollte präoperativ immer ein CT-Scan veranlasst werden. Es kann sinnvoll sein, die Operation navigiert durchzuführen.

### Mikrobiologie

Aufgrund der Tatsache, dass PPT in den meisten Fällen auf einer Infektion der oberen Atemwege beruhen, spielen Streptokokken (alpha- und beta-hämolytische Streptokokken), Haemophilus influenzae, Staphylococcus aureus und andere Anaerobier (Fusobacterium- und Bacteroides-Arten) eine entscheidende Rolle bei der Pathogenese [12]. In den hier vorgestellten Fällen wurde Streptococcus intermedius bei drei von vier Patienten nachgewiesen und Staphylococcus capitis bei einem Patienten (Tab. 1). Streptococcus intermedius ist für sein hohes pathogenes Potenzial bekannt, und es wird berichtet, dass es bei Patienten mit gleichzeitiger Sinusitis sowie bei Patienten mit mehreren Risikofaktoren eine erhöhte Assoziation mit intrakraniellen Abszessen gibt [22]. Im Gegensatz dazu waren die hier vorgestellten Patienten jung, gesund und immunkompetent. Die meist frühe und kalkulierte Antibiotikatherapie macht Komplikationen bei akuter Sinusitis immer seltener. Bei langen und komplizierten Verläufen einer Sinusitis sowie beim Auftreten einer entsprechenden Symptomatik nach kürzlich durchgeführter chirurgischer Intervention sollte eine Resistenztestung mit angepasster antibiotischer Therapie erfolgen [22].

Die Resistenztestung von Streptococcus intermedius zeigte eine antimikrobielle Empfindlichkeit gegen Penicillin, Ampicillin, Cefotaxim, Ceftriaxon und Vancomycin. Für Staphylococcus capitis wurde eine antibiotische Empfindlichkeit gegenüber Flucloxacillin, Vancomycin, Ciprofloxacin, Doxycyclin und Co-Trimoxazol gefunden.

### Behandlung

Der Goldstandard der Therapie des PPT ist die kalkulierte Antibiotikatherapie in Kombination mit einer operativen Drainage des Infekts. Eine schnelle und wirksame Behandlung ist von entscheidender Bedeutung, um weitere Komplikationen wie epidurale oder intrazerebrale Komplikationen zu vermeiden. Somit sollte die Antibiotikabehandlung bereits bei klinischem Verdacht auf PPT begonnen und nach einer entsprechenden Erregerdiagnostik an das nachgewiesene Spektrum angepasst werden. Die Erstbehandlung sollte mit einem zentral wirksamen Breitbandantibiotikum, wie beispielsweise mit Ceftriaxon, welches auch in unseren Fällen angewendet wurde, erfolgen [14]. Die wichtigste Säule der Therapie ist jedoch die chirurgische Drainage des Abszesses. Die Art der gewählten Operation hängt von der individuellen Anatomie und dem Ausmaß der Infektion ab. Es besteht die Möglichkeit eines endonasalen als auch eines offenen Zugangs zur Stirnhöhle oder der Kombination von beiden. Eine intrakranielle Abszessbildung erfordert eine zusätzliche neurochirurgische Behandlung [12]. Die externe Drainage hat den entscheidenden Vorteil, dass der gesamte Bereich der Stirnhöhle eingesehen und der von Osteomyelitis betroffene Knochen entfernt werden kann und die Stirnhöhle bestmöglich drainiert wird [4, 11]. Im Gegensatz dazu ist FESS eine minimal-invasive Technik und bietet die Möglichkeit einer gewebeschonenden Drainage. Hier kann im Gegensatz zum Zugang von außen der ostiomeatale Komplex als engste Drainagestelle des Nasennebenhöhlensystems sowie der Recessus frontalis als entscheidende Struktur für die Stirnhöhlendrainage beurteilt und behandelt werden. Neben dem Vorteil fehlender äußerer Narben haben Patienten nach FESS im Gegensatz zu einem offenen Ansatz eine deutlich verkürzte Rekonvaleszenz und damit eine kürzere Krankenhausaufenthaltszeit. Eine isolierte Drainage des subperiostalen Abszesses scheint ebenfalls mit einer erhöhten Rezidivrate oder weiteren Komplikationen verbunden zu sein [11, 13]. Abhängig von diesen Erkenntnissen sollten chirurgische Therapien und die Rehabilitation in enger Zusammenarbeit zwischen den verschiedenen Fachdisziplinen HNO-Heilkunde, Neurochirurgie, Radiologie, Augenheilkunde, Mikrobiologie/Infektionskrankheiten und Pädiatrie erfolgen. Eine alleinige Antibiotikatherapie ohne operative Behandlung scheint mit einer hohen Rezidivrate verbunden zu sein und kann nach heutigem Forschungsstand eine chirurgische Therapie nicht ersetzen [16]. In den hier beschriebenen Fällen wurde die Drainage der Stirnhöhle stets von endonasal durchgeführt. Diese wurde einmal mit einer Drainage der Orbita von außen kombiniert, und ein anderes Mal wurde ein präfrontaler Abszess über die Korrugatorfalte entlastet. In drei der Fälle wurden die Patienten zusätzlich mit einer Kraniotomie in Kombination mit einem externen Zugang zur Behandlung der subgalealen und epiduralen sowie intraduralen Abszessformationen therapiert (Tab. 1).

## Fazit für die Praxis

Der Pott-Puffy-Tumor bleibt immer noch eine seltene Komplikation einer akuten oder chronischen Sinusitis, insbesondere bei kleinen Kindern.

Hier ist die konsequente Behandlung einer Sinusitis, auch im Säuglingsalter bei einem noch nicht vollständig pneumatisierten Nasennebenhöhlensystem, zur Vermeidung von intrakraniellen Komplikationen von entscheidender Bedeutung, da es ggf. zu lebensbedrohlichen Komplikationen kommen kann.

Die hier vorgestellten Fälle unterstreichen die Wichtigkeit einer engen interdisziplinären Zusammenarbeit verschiedener Fachdisziplinen, um langfristige Komplikationen zu vermeiden und eine vollständige Rehabilitation zu unterstützen.
